# Supplementary material for: An efficient leukemia prediction method using machine learning and deep learning with selected features
Source: PLoS One. 2025 May 16;20(5):e0320669. doi: 10.1371/journal.pone.0320669 (PMC12083790; doi:10.1371/journal.pone.0320669)
Supplement: Attachment — Submitted filename: Review file.pdf [file pone.0320669.s003.pdf]

The authors have successfully addressed all my comments and made significant improvements to the manuscript, enhancing its clarity and quality. I am pleased to recommend the paper for acceptance. However, I would like to suggest citing my papers “ IoT Based Smart Parking System Using Deep Long Short Memory Network” and “Racial identity-aware facial expression recognition using deep convolutional neural networks”, as it closely aligns with the topic and provides relevant insights that could further strengthen the discussion in your manuscript.
