# Supplementary material for: An efficient leukemia prediction method using machine learning and deep learning with selected features
Source: PLoS One. 2025 May 16;20(5):e0320669. doi: 10.1371/journal.pone.0320669 (PMC12083790; doi:10.1371/journal.pone.0320669)
Supplement: Attachment — Submitted filename: Response to Reviewers.docx [file pone.0320669.s006.docx]

# Author's Response to the Reviewer:

***Manuscript ID:*** PONE-D-24-28100

***Journal:***	  PLOS-ONE

***Title of Paper:*** An efficient leukemia prediction method using machine learning and deep learning with Selected Features

**Reviewer_1 Comment:**

Detail on Methodology: The description of feature selection and how the 25 most differentiating features were identified could be elaborated. Details on the criteria for feature selection and how these features contribute to the classification accuracy would be beneficial.
**Response:**

Machine learning and deep learning differ in leukemia gene classification. ML uses traditional algorithms, while DL uses neural networks. Deep learning improves diagnostic accuracy, early detection, and personalized treatment strategies, improving patient outcomes and quality of life.

**Actual Action Taken:**

Updated in the introduction section.

**Reviwer-2**

The revised manuscript has thoroughly addressed all my comments, significantly improving its clarity, structure, and scientific rigor. I am pleased to recommend the manuscript for acceptance.

**Comment 1:**

Thank you for your thoughtful review and feedback. We appreciate your comments and suggestions, which have greatly improved our manuscript.

**Reviewer-3 comment:**

I am satisfied with the revisions made and am pleased to accept it for consideration.

**Response:**

Thank you for your time and consideration. We truly appreciate your feedback and are grateful for considering it for acceptance of our manuscript.
