# Supplementary material for: An efficient leukemia prediction method using machine learning and deep learning with selected features
Source: PLoS One. 2025 May 16;20(5):e0320669. doi: 10.1371/journal.pone.0320669 (PMC12083790; doi:10.1371/journal.pone.0320669)
Supplement: Attachment — Submitted filename: Reviwer comments plus one 021825.docx [file pone.0320669.s005.docx]

**An efficient leukemia prediction method using machine learning and deep learning with Selected Features**

Manuscript presents a promising approach for leukemia prediction and classification using machine learning (ML) and deep learning (DL) techniques with selected features from CuMiDa gene expression data. The study is relevant to cancer diagnostics and contributes valuable insights into feature selection, classification accuracy, and deep learning performance.

I would like to commend you on your excellent work in predicting and classifying leukemia subtypes using machine learning (ML) and deep learning (DL) techniques. Your manuscript presents an innovative approach by utilizing the CuMiDa dataset and effectively selecting the top features for classification. The proposed methodology and results are promising and demonstrate the potential for enhancing leukemia diagnosis and treatment.

Overall, the manuscript demonstrates innovative use of various techniques and presents promising results in leukemia prediction. This paper has the potential to make a significant contribution to the field of cancer diagnostics.

Recommendation: Accept

Dr. Biba Vikas
